# Effect of Comorbidity Assessed by the Charlson Comorbidity Index on the Length of Stay, Costs and Mortality among Older Adults Hospitalised for Acute Stroke

**DOI:** 10.3390/ijerph15112532

**Published:** 2018-11-12

**Authors:** Richard Ofori-Asenso, Ella Zomer, Ken Lee Chin, Si Si, Peter Markey, Mark Tacey, Andrea J. Curtis, Sophia Zoungas, Danny Liew

**Affiliations:** 1 Centre of Cardiovascular Research and Education in Therapeutics, Department of Epidemiology and Preventive Medicine, Monash University, Melbourne, VIC 3004, Australia; richard.ofori-asenso@monash.edu (R.O.-A.); ella.zomer@monash.edu (E.Z.); ken.chin@monash.edu (K.L.C.); si.si@monash.edu (S.S.); mark.tacey@monash.edu (M.T.); 2 Epidemiological Modelling Unit, Department of Epidemiology and Preventive Medicine, Monash University, Melbourne, VIC 3004, Australia; 3 Division of Metabolism, Ageing and Genomics, Department of Epidemiology and Preventive Medicine, Monash University, Melbourne, VIC 3004, Australia; andrea.curtis@monash.edu (A.J.C.); sophia.zoungas@monash.edu (S.Z.); 4 Alfred Hospital, Melbourne, VIC 3004, Australia; P.Markey@alfred.org.au

**Keywords:** stroke, cerebrovascular disease, comorbidity, cost, hospitalisation

## Abstract

The burden of comorbidity among stroke patients is high. The aim of this study was to examine the effect of comorbidity on the length of stay (LOS), costs, and mortality among older adults hospitalised for acute stroke. Among 776 older adults (mean age 80.1 ± 8.3 years; 46.7% female) hospitalised for acute stroke during July 2013 to December 2015 at a tertiary hospital in Melbourne, Australia, we collected data on LOS, costs, and discharge outcomes. Comorbidity was assessed via the Charlson Comorbidity Index (CCI), where a CCI score of 0–1 was considered low and a CCI ≥ 2 was high. Negative binomial regression and quantile regression were applied to examine the association between CCI and LOS and cost, respectively. Survival was evaluated with the Kaplan–Meier and Cox regression analyses. The median LOS was 1.1 days longer for patients with high CCI than for those with low CCI. In-hospital mortality rate was 18.2% (22.1% for high CCI versus 11.8% for low CCI, *p* < 0.0001). After controlling for confounders, high CCI was associated with longer LOS (incidence rate ratio [IRR]; 1.35, *p* < 0.0001) and increased likelihood of in-hospital death (hazard ratio [HR]; 1.91, *p* = 0.003). The adjusted median, 25th, and 75th percentile costs were AUD$2483 (26.1%), AUD$1446 (28.1%), and AUD$3140 (27.9%) higher for patients with high CCI than for those with low CCI. Among older adults hospitalised for acute stroke, higher global comorbidity (CCI ≥ 2) was associated adverse clinical outcomes. Measures to better manage comorbidities should be considered as part of wider strategies towards mitigating the social and economic impacts of stroke.

## 1. Introduction

Non-communicable chronic diseases are prevalent among older people [1]. Data from over 60 million older adults (aged ≥65 years) in 30 high-income countries showed that 88% (interquartile range [IQR] 80.8–93.2) had at least one chronic medical condition (CMC), and more than 66% had multimorbidity (i.e., the presence of two or more CMCs) [2].

Multimorbidity is associated with higher utilisation of healthcare services and polypharmacy and places a significant burden on the healthcare system [3]. A poor understanding of multimorbidity partly contributes to the suboptimal treatment of chronic diseases worldwide [4].

Cerebrovascular diseases, including stroke, are important causes of global morbidity and mortality. In 2013, stroke was the second most common cause of death (11.8% of all deaths or 6.5 million deaths) and the third most common cause of disability worldwide (4.5% of Disability-Adjusted Life Years [DALYs] from all causes) [5]. In Australia, stroke death rates declined by 70% from 1970 to 2010 [6]. Despite this, stroke is still the third leading cause of death, being responsible for 10,869 deaths (6.8% of all deaths) among Australians in 2015. In 2017, an estimated 475,160 people (~2% of Australians), were living with stroke; over 70% were persons aged 65 years and over [7]. Consequently, stroke exerts a considerable economic burden [8]; the total financial cost of stroke in Australia was estimated to exceed AUD$5 billion in 2012 [9].

Up to 90% of stroke sufferers have one or more comorbid conditions and almost a quarter have ≥5 CMCs [10,11]. Current healthcare services are not adequately designed to cater for stroke patients with comorbidities [12]. Furthermore, the impact of comorbidities on outcomes among stroke patients is poorly understood [13]. Studies that have examined the impact of comorbidities on survival among older stroke patients are limited by small sample sizes [14,15,16]. Importantly, the specific impact of comorbidities on healthcare utilisation and costs among older adults hospitalised for acute stroke has not been adequately quantified within the Australian context.

The Charlson Comorbidity Index (CCI) has been demonstrated in several studies to be useful for predicting the prognosis of real-world patients with comorbidities [17,18]. Higher CCI scores are correlated with poorer functional status at discharge and an increased risk of one-year mortality in older stroke patients [19]. In the present study on older adults hospitalised for acute stroke, we sought to evaluate whether or not higher comorbidity burden, as measured by CCI, is associated with increased length of hospital stay (LOS), hospital costs, and death rates.

## 2. Materials and Methods

### 2.1. Study Design and Population

We undertook a retrospective analysis of the records of all patients admitted for acute stroke between 1 July 2013 and 31 December 2015 to the Alfred Hospital, a large tertiary hospital with an established stroke unit in Melbourne, Victoria. Stroke was defined by the International Classification of Diseases, Version 10, Australian Modification (ICD-10-AM) codes [20], comprising intracerebral haemorrhage = I61.0, I61.1, I61.2, I61.3, I61.4, I61.5, I61.6, I61.8, I61.9, I62.9; unspecified non-traumatic intracranial haemorrhage = I62.9; cerebral infarction = I63.0, I63.1, I63.2, I63.3, I63.4, I63.5, I63.6, I63.8, I63.9; and undetermined stroke (not specified as haemorrhage or infarction) = I64. Patients aged 65 years and over were included in the analysis if any of the above events were documented as their primary reason for admission.

### 2.2. Outcomes

The study outcomes were in-hospital death, length of stay (LOS), and hospital costs. Hospital mortality rate was defined as the ratio of deaths to the total number of admitted cases. The LOS was calculated as the period from the admission date to the date of separation (death or discharged alive). The costs of each admission were determined using the assigned Weighted Inlier Equivalent Separation (WIES) value. WIES values are used by the Victorian Government to determine casemix funding for hospitals [21]. WIES is allocated according to Diagnosis Related Group (DRG), LOS, and a number of variable co-payments. In the 2017/2018 financial year, a unit of WIES was valued at AUD$4732 and AUD$3544 for public and private patients, respectively.

### 2.3. Covariates

Demographic data of interest included the patients’ date of birth, sex, place of birth, usual residence, postcode of residence, marital status, and Aboriginal and Torres Strait Islander status. Postcode of residence was used to reflect quintile of socio-economic status as per the Australian Bureau of Statistics (ABS) Socio-Economic Indices for Areas (SEIFA) and the Index of Relative Socio-Economic Advantage and Disadvantage (IRSAD). Clinical data included the type of stroke, quality of care indicators (e.g., treatment carried out in a stroke unit), presence or absence of intensive care unit (ICU) stay, and complications occurred during the hospital stay.

For each patient, we calculated the CCI based on 17 comorbid conditions: congestive heart failure (weight = 1), myocardial infarct (weight = 1), cerebrovascular disease (weight = 1), chronic pulmonary disease (weight = 1), paraplegia (weight = 2), dementia (weight = 1), diabetes without complications (weight = 1), diabetes with complications (weight = 2), cancer (weight = 2), metastatic cancer (weight = 6), mild liver disease (weight = 1), moderate or severe liver disease (weight = 3), peptic ulcer disease (weight = 1), peripheral vascular disease (weight = 1), rheumatologic disease (weight = 1), renal disease (weight = 2), and human immunodeficiency virus [HIV]/acquired immune deficiency syndrome [AIDS] (weight = 6) [22]. In addition, we collected data on other comorbidities such as hypertension and atrial fibrillation, which were not incorporated in the CCI calculation.

## 3. Statistical Analyses

Descriptive statistics were applied to summarise patient characteristics. Variables were compared across patients in the two CCI categories using the *χ*² test for proportions, Student *t*-test for means, and Kruskal–Wallis rank test for the comparison of medians. Kaplan–Meier survival curves and log-rank test were used to compare in-hospital mortality between groups. A Cox proportional hazards model was then derived and adjusted for baseline clinical and sociodemographic parameters and year of admission. The test of proportional hazards assumption was performed for model calibration. In the multivariable Cox proportional hazards model, the only variable violating the proportional hazards assumption was stroke type, and therefore this was included as a stratifying factor [23]. The relationship between CCI and LOS was evaluated via a negative binomial regression (NBR) model (because of over-dispersion of the LOS data) [24], while controlling for the same list of covariates listed above. Relative LOS were described in terms of incidence-rate ratios (IRRs). Quantile regression, which permits the assessment of the effects of a covariate on all parts of the cost distribution (i.e., upper, lower, and median) rather than just the mean, as with an ordinary least squares (OLS) model [25], was used to analyse the relationship between hospital costs and CCI. An ordinary least squares (OLS) model adopting a backward stepwise approach (with variable selection cut off values of *p* < 0.10) was used to identify predictors to be included in the quantile regression model. In all analyses, CCI was categorised as low [CCI 0–1] and high [CCI ≥ 2] [19]. Unless otherwise specified, *p*-values < 0.05 were considered significant. All statistical analyses were performed with STATA (version 15/IC, Stata Corp, College Station, TX, USA).

## 4. Ethics Approval

The study received approval from the Alfred Hospital Human Research Ethics Committee.

## 5. Results

### 5.1. Cohort Characteristics

A total of 776 older adults was hospitalised for acute stroke between 1 July 2013 and 31 December 2015, of whom 66.2%, 27.3%, and 6.5% had ischaemic, haemorrhagic, and undetermined stroke, respectively. The mean age of the cohort was 80.1 (SD ± 8.3) years and 46.7% were female. More than half (50.4%) were married or in a de-facto relationship. Approximately 3.0% were patients from an aged care residential facility. Overall, 48.6% were born outside of Australia and 10.6% were non-English-speaking and required an interpreter. Less than 1% of the cohort identified themselves as Aboriginal or Torres Strait Islander. Similarly, <1% were admitted as private patients. The percentage of the study cohort with hypertension, atrial fibrillation, congestive heart failure, metastatic cancer, renal disease, diabetes with complications, or current smokers was 65.3%, 24.2%, 6.7%, 3.4%, 10.7%, 10.4%, and 3.2%, respectively. The mean CCI of the cohort was 2.21 (SD ± 2.2) and the median was 2.0 (IQR 0–3). The percentage of patients with high CCI score (≥2) was 61.9%. One in ten (10.3%) of the patients were admitted to the ICU. Table 1 summarises the patients’ demographic and clinical characteristics according to low or high CCI groups. Both groups were similar in most characteristics except that the low CCI group had more haemorrhagic strokes while patients with a high CCI score experienced more complications during hospital stay.

### 5.2. Length of Stay

**Figure 1:**
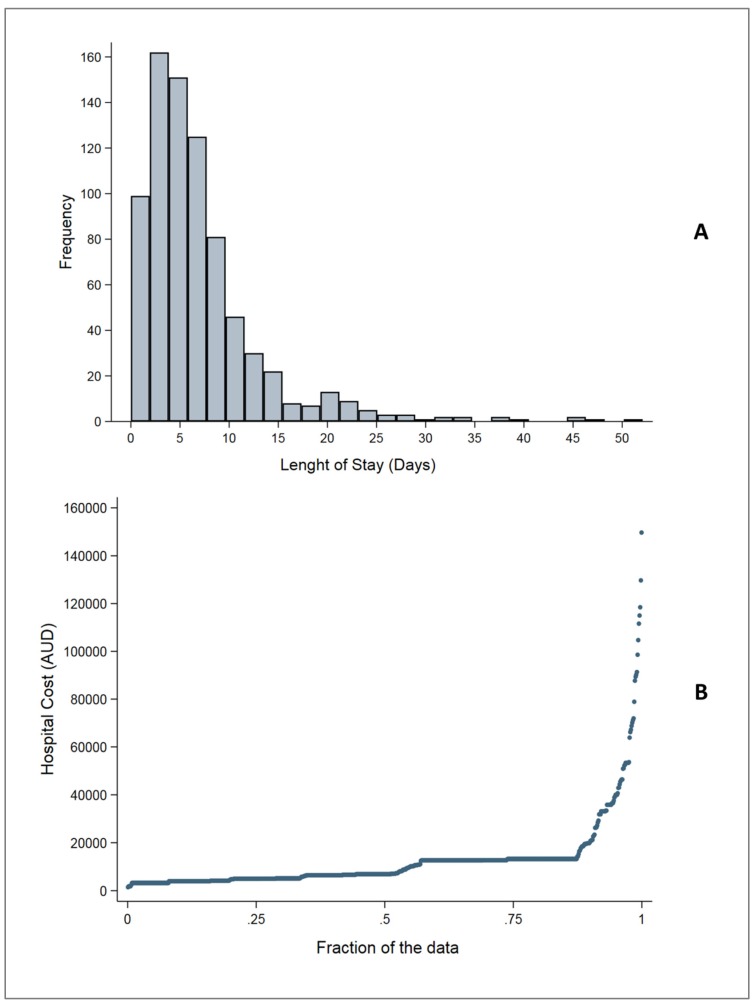
(**A**) Histogram depicting the distribution of LOS; and (**B**) quantile distribution of hospital costs among the study population of older adults hospitalised for acute stroke.

Figure 1A depicts LOS among the study population. The median LOS was 5.44 (IQR 3.0–8.69) days. The unadjusted median LOS was similar among patients with ischaemic (5.67, IQR 3.33–8.75) and haemorrhagic stroke (5.63, IQR 2.85–9.08); *p*-value for difference = 0.6757. For undetermined stroke, the median LOS was much lower (3.0, IQR 1.79–5.50). The median LOS in patients with a low CCI (0–1) was 4.81 (IQR 2.85–7.71) days compared to 5.88 (IQR 3.27–9.44) days in patients with a high CCI (≥2) (*p*-value for difference = <0.001). After adjusting for potential confounders in the NBR model, a high CCI score was associated with a 35% higher likelihood of increased LOS compared to a low CCI score (IRR 1.35, 95% CI 1.21 to 1.49; *p* < 0.001).

### 5.3. Costs

The hospital cost among the cohort varied widely from AUD$1470 to $149,637. Figure 1B illustrates the quantile distribution of direct hospital costs incurred by the study cohort. The overall unadjusted median hospital cost among the cohort was AUD$6924 (IQR $4971–$13,238). The unadjusted median cost among patients with ischaemic stroke was AUD$7022 (IQR $5112–$13,237) and that among those with haemorrhagic stroke was AUD$8026 (IQR $4139–$13,238); *p*-value for difference = 0.8155. The unadjusted median cost among patients with undetermined stroke was much lower (AUD$5112, IQR $3943–$7032). Patients with a low CCI score (0–1) incurred lower median unadjusted hospital costs (AUD$6447; IQR $4971–$12,658) compared to those with a high CCI score (≥2) (AUD $12,658; IQR $5536–$13,238) (*p* = 0.0001). When adjusted for potential confounders (sex, separation status (died or discharged alive), ICU stay, and treatment within stroke unit), the median hospital cost was AUD$2481 (26.1%), and 25th and 75th percentile costs were AUD$1446 (28.1%) and AUD$3140 (27.9%), respectively, which was higher in the high CCI (≥2) group compared to the low CCI group (Table 2, models 1, 2, and 3).

### 5.4. Mortality

Overall, in-hospital death occurred in 18.2% of the study cohort. The unadjusted in-hospital mortality rate varied by stroke sub-type: 12.7% in patients with ischaemic stroke and 30.2% in those with haemorrhagic stroke (*p*-value for differences = <0.001). The unadjusted proportion of patients with undetermined stroke who died was 24.0%. The proportions of patients with a low CCI score (0–1) and a high CCI score (≥2) who died were 11.8% and 22.1%, respectively (*p*-value for difference in unadjusted proportions = <0.0001).

**Figure 2:**
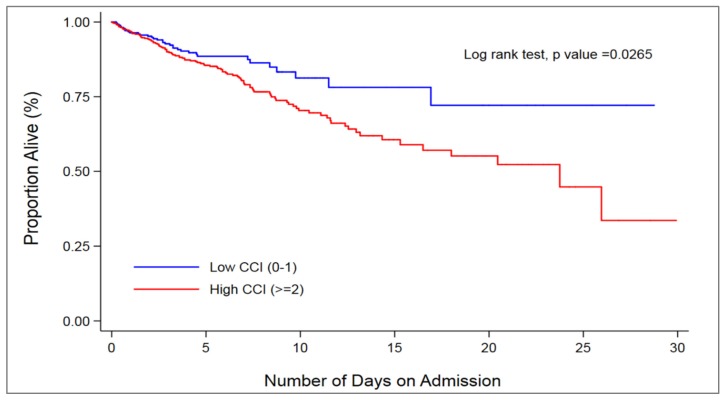
Unadjusted Kaplan–Meier survival curves for patients admitted for acute stroke stratified by Charlson Comorbidity Index (CCI) categories.

Patients with low CCI scores had better survival rates compared to patients with high CCI scores (*p*-value for rank test = 0.0265) (Figure 2). A high CCI score (≥2) was associated with nearly two times greater likelihood of death than a low CCI score (0–1) (hazard ratio [HR]; 1.91, 95% CI 1.25–2.90, *p* = 0.003) after adjusting for key clinical and sociodemographic confounders as listed in Table 1.

## 6. Discussion

In the present study, we found that a high CCI score was associated with increased LOS, higher hospital costs, and a greater likelihood of in-hospital death. Specifically, patients with a high CCI score (≥2) had a 1.1 day longer LOS (35% increased risk) compared to those with a low CCI score (0–1). The adjusted median hospital cost for patients with high CCI was 26.1% (AUD$2481) higher than for patients with low CCI. A high CCI score was also associated with 91% increased risk of death compared to a low CCI score.

Few studies have examined the relationship between comorbidities and LOS, mortality, and, in particular, healthcare costs among older adults hospitalised for acute stroke. Our study highlighted the substantive clinical and economic impact of comorbidities among a diverse population of older adults from a developed country with a universal healthcare system.

Globally, older adults have a higher risk of stroke mortality than younger individuals [26,27,28,29]. Grube and colleagues reported that persons aged ≥85 years were more likely to die after stroke compared with those aged <65 years (odds ratio [OR]; 13.50, 95% CI; 5.54–32.89) [26]. In our study, almost one in five (18.2%) of the cohort experienced in-hospital death, which was higher compared to that reported among cohorts of older adults in Italy (10.9%) [14], China (3.1%) [30], Saudi Arabia (11.0%) [31], and Canada (13.4%) [32], although a recent study reported a much higher (35.7%) in-hospital stroke death rate in Turkey [33]. The differences were likely to be due to a myriad of factors, including study design, disease definition, local factors (e.g., the standard of hospital care or treatment received), but particularly due to the fact that, in the other studies, all patients had ischaemic stroke, for which the case fatality was significantly lower than for haemorrhagic stroke [34]. Among our cohort, the unadjusted death rates among those with haemorrhagic stroke was over two times higher than those with ischaemic stroke. Our study population was also older than the populations from the other studies. Furthermore, the differences could be attributed to acuity and accuracy of admission diagnoses for stroke in Australia or issues with classification. For example, in areas with less experience of using ICD coding, some transient ischaemic attacks (TIAs) with better outcomes could be misclassified as stroke [35].

The ageing process, as represented by chronically elevated pro-inflammatory markers, is a leading risk factor for major CMCs such as cardiovascular diseases, dementia, and chronic kidney disease [1]. The CCI is reflective of the cumulative burden of disease. We found that CCI was strongly associated with in-hospital death among hospitalised acute stroke patients, supporting the use of CCI as a tool in predicting in-hospital death among this population [17,36]. CCI has been consistently shown to independently predict death among patients with sepsis [37], congestive heart failure, and cancer [38].

Our results also indicate that CCI is associated with longer LOS and higher hospitalisation costs in Australia. CCI has previously been found to predict LOS in stroke patients [39], as well as in patients with cancer [38], sepsis, or those undergoing surgical procedures [40]. Among patients hospitalised for hip fracture in the USA, Johnson and colleagues [41], observed that patients with a CCI score of 2, on average, stayed 1.92 extra days in the hospital and incurred USD$8698 extra costs compared to those with a CCI score of 0.

The relationship between the CCI score and multiple outcomes (cost, LOS, and mortality) as observed in this study supports the notion that CCI can be adapted for risk stratification, particularly when negotiating bundled payments [41,42], as it embodies a good representation of the complexity and current state of the patient [14]. For clinicians working within acute care settings, CCI could be employed to identify patients most likely to experience worse outcomes for whom greater monitoring may be required. Given the clinical and economic impact of comorbidities among older adults with acute stroke, measures to manage comorbidities are important and should be considered as part of the wider strategies aimed at reducing the social and economic impacts of stroke. For example, systematic evaluation and screening of comorbidities may allow for earlier identification of patients at risk and targeting of in-hospital management. Improved care coordination, inter-disciplinary transition of care, and management counselling during hospitalisation are all important strategies that could contribute to the optimised management of comorbidities [3,43].

There were several limitations to our study that warrant mention. First, we analysed only administrative hospital data that were not collected for research purposes. The data may be subject to coding errors or omissions and inaccurate classification of diagnoses and complications. Secondly, information was not available for important covariates such as body mass index (BMI), disease severity, and laboratory and imaging results. Thirdly, given that our sample population was selected from a single hospital, selection bias may limit the generalisability of our findings. Fourthly, the CCI did not account for the effects of gradations of disease progression and severity. Furthermore, because of the differences in our study population and that from which the original CCI was developed, the performance of the CCI as a prognostic variable might not be the same [44]. Finally, the use of in-hospital data meant that we were unable to examine the impact of CCI on longer-term post-discharge outcomes.

## 7. Conclusions

Among older adults hospitalised for acute stroke, high global comorbidity (CCI ≥ 2) was associated with increased LOS, costs, and mortality. The adaptation of CCI within acute care settings has the potential to offer a rapid and reliable approach to identifying patients likely to experience adverse outcomes who may benefit from closer monitoring. Measures to address comorbidities need to be considered as part of wider strategies for reducing the social and economic impact of stroke.

## Figures and Tables

**Table 1:** Sociodemographic and clinical characteristics of older adults hospitalised for acute stroke.

Variables	All (*n* = 776)	CCI ^§^		*p*-Value ^†^
		Low (*n* = 296)	High (*n* = 480)	
Mean age, years (SD)	80.1 (8.3)	79.7 (8.5)	80.3 (8.2)	0.123
≥85 years, *n* (%)	265 (34.2)	89 (30.1)	176 (36.7)	0.170
Female, *n* (%)	362 (46.7)	144 (48.7)	218 (45.4)	0.381
Country of birth, *n* (%)				
Australia	399 (51.4)	172 (58.1)	223 (47.3)	0.031
Asia	37 (4.8)	11 (3.7)	26 (5.4)	
Europe	251 (32.3)	82 (27.7)	169 (35.2)	
Other	86 (11.5)	31 (10.5)	58 (12.1)	
Interpreter required, *n* (%)	82 (10.6)	27 (9.1)	55 (11.5)	0.304
Married or in a de facto relationship, *n* (%)	391 (50.4)	154 (51.4)	239 (49.8)	0.361
Type of stroke, *n* (%)				
Haemorrhagic	212 (27.3)	102 (34.4)	110 (22.9)	0.001
Ischaemic	514 (66.2)	174 (58.8)	340 (70.8)	
Undetermined	50 (6.4)	20 (6.8)	30 (6.3)	
Patient with multiple records, *n* (%)	34 (4.4)	12 (4.1)	22 (4.6)	0.726
Comorbidities, *n* (%)				
Hypertension	507 (65.3)	192 (64.9)	315 (65.6)	0.829
Diabetes with complication	81 (10.4)	0 (0.0)	81 (16.9)	<0.001
Metastatic cancer	26 (3.4)	0 (0.0)	26 (5.4)	<0.001
Atrial fibrillation	188 (24.2)	64 (21.6)	124 (25.8)	0.183
Renal disease	83 (10.7)	0 (0.0)	83 (17.3)	<0.001
Congestive heart failure	52 (6.7)	5 (1.7)	47 (9.8)	<0.001
Dementia	35 (4.5)	13 (4.4)	22 (4.6)	0.901
Chronic pulmonary disease	25 (3.2)	7 (2.4)	18 (3.8)	0.288
Myocardial infarction	49 (6.3)	9 (3.0)	40 (8.3)	0.003
Smoking (current), *n* (%)	25 (3.2)	9 (3.0)	16 (3.3)	0.822
Treated in a stroke unit, *n* (%)	480 (61.9)	171 (57.8)	309 (64.4)	0.066
Admitted to ICU, *n* (%)	80 (10.3)	29 (9.8)	51 (10.6)	0.713
Developed complication, *n* (%)	463 (59.7)	147 (49.7)	316 (65.8)	<0.001
Patient from aged care residential facility, *n* (%)	23 (3.0)	6 (2.0)	17 (3.5)	0.372
IRSAD, *n* (%)				
Quintile 1 (most disadvantaged)	163 (21.0)	75 (25.3)	88 (18.4)	0.031
Quintile 2	160 (20.6)	59 (19.9)	101 (21.0)	
Quintile 3	196 (25.2)	65 (22.0)	131 (27.3)	
Quintile 4	127 (16.4)	30 (13.5)	87 (18.1)	
Quintile 5 (least disadvantaged)	130 (16.8)	57 (19.3)	73 (15.2)	
Admission year, *n* (%)				
2013	187 (24.1)	74 (25.0)	113 (23.5)	0.885
2014	284 (36.6)	106 (35.8)	178 (37.1)	
2015	305 (39.3)	116 (39.2)	189 (39.4)	

*n* = number of patients; SD = standard deviation; CCI = Charlson Comorbidity Index; ICU = intensive care unit; IRSAD = Index of Relative Socio-Economic Advantage and Disadvantage; ^†^ differences between proportions were assessed via the chi-square test and means were assessed with the Student *t*-test; ^§^ low CCI (0–1) and high CCI (≥2).

**Table 2:** Regression models of adjusted median and percentile costs associated with hospitalised cases of acute stroke.

	Model 1 ^a^: 25th Percentile		Model 2 ^a^: Median		Model 3 ^a^: 75th Percentile	
	Estimate	95% CI	Estimate	95% CI	Estimate	95% CI
High CCI (≥2)	1446	832–2060 **	2483	788–4175 **	3140	1214–5068 **
Constant	5148	4067–6229 **	9521	6539–12,501 **	11,257	7865–14,650 **

^a^ Adjusted for sex, separation status (i.e., died or discharged alive), intensive care unit stay, and treatment within stroke unit; ** statistically significant at *p* < 0.01.
